# Association of the *RNF213* p.R4810K Variant With the Outer Diameter of Cervical Arteries in Patients With Ischemic Stroke

**DOI:** 10.1161/SVIN.121.000298

**Published:** 2022-03-24

**Authors:** Eriko Yamaguchi, Takeshi Yoshimoto, Shiori Ogura, Kozue Saito, Satoshi Saito, Yorito Hattori, Kazuo Wasida, Kunihiro Nishimura, Kazunori Toyoda, Masatoshi Koga, Masafumi Ihara

**Affiliations:** ^1^ Department of Neurology National Cerebral and Cardiovascular Center 6‐1, Kishibe‐Shimmachi Suita Osaka 5648565 Japan; ^2^ Department of Cerebrovascular Medicine National Cerebral and Cardiovascular Center 6‐1, Kishibe‐Shimmachi Suita Osaka 5648565 Japan; ^3^ Department of Neurology Nara Medical University 840 Shijo‐cho Kashihara Nara 6348521 Japan; ^4^ Department of Preventive Medicine and Epidemiologic Informatics National Cerebral and Cardiovascular Center 6‐1, Kishibe‐Shimmachi Suita Osaka 5648565 Japan

**Keywords:** cervical artery, ischemic stroke, outer diameter, *RNF213* variant

## Abstract

**Background:**

We investigated the impact of the p.R4810K variant of *RNF213* (ring finger protein 213) gene, a susceptibility gene of moyamoya disease in East Asia, on the outer diameter of cervical parts of carotid and vertebral arteries (VAs).

**Methods:**

We examined consecutive Japanese patients with ischemic stroke who underwent carotid ultrasonography between 2015 and 2019. Patient background and the carotid ultrasonography‐measured outer diameter of extracranial cervical arteries, including the common carotid artery, internal carotid artery, external carotid artery, and cervical VA, were compared between variant carriers and noncarriers. Outer diameters of each artery were defined as the mean distance from far to near wall adventitia of right and left target arteries using carotid ultrasonography. The average diameter of both cervical portions of common carotid arteries, internal carotid arteries, external carotid arteries, and the dominant side diameter of both cervical VAs were used.

**Results:**

Of the 617 adult patients (204 women; median age, 74 years) analyzed, 26 (4.2%) carried the *RNF213* p.R4810K variant. Variant carriers were significantly younger (*P*<0.01) and had more frequent steno‐occlusion of the M1 segment of the middle cerebral artery (*P*<0.01). Multivariate logistic regression analysis showed that variant carriers had significantly smaller mean diameters in the common carotid artery (7.25 versus 8.22 mm; adjusted odds ratio [aOR] per 1 mm decrease, 2.94; 95% CI, 1.69–5.00), cervical internal carotid artery (4.99 versus 5.55 mm; aOR, 1.66; 95% CI, 1.03–2.70), and cervical VA (3.55 versus 4.10 mm; aOR, 2.56; 95% CI, 1.33–4.76) than noncarriers. Mean diameters of the common carotid artery (aOR, 3.44; 95% CI, 2.08–5.88) and cervical internal carotid artery (aOR, 2.04; 95% CI, 1.23–3.33) and the dominant diameter of the cervical VA (aOR, 3.23; 95% CI, 1.72–5.88) were also smaller in variant carriers even when the analysis was restricted to patients without bilateral steno‐occlusion in target vessels or intracranial arteries distal to target vessels.

**Conclusion:**

*RNF213* p.R4810K variant carriers have smaller cervical arterial outer diameters in both anterior and posterior circulations than noncarriers with ischemic stroke.

**Registration:**

URL: https://www.clinicaltrials.gov; Unique identifier: NCT02251665.

**Nonstandard Abbreviations and Acronyms**
BA: basilar artery
CCA: common carotid artery
CUS: carotid ultrasonography
ICA: internal carotid artery
IS: ischemic stroke
MCA: middle cerebral artery
MMD: moyamoya disease
MR: magnetic resonance
NCVC: National Cerebral and Cardiovascular Center
*RNF213*
: ring finger protein 213
VA: vertebral artery

**Clinical Perspective**

- Adult patients with ischemic stroke with the *RNF213* (ring finger protein 213) gene p.R4810K variant show small outer diameters of cervical arteries in both anterior and posterior circulations.
- The *RNF213* p.R4810K variant is involved not only in moyamoya disease characterized by intracranial artery stenosis but also in general ischemic stroke accompanied by small diameters of extracranial cervical arteries.

The p.R4810K variant (c.14576G>A) of the *RNF213* (ring finger protein 213) gene has been identified as a susceptibility gene for moyamoya disease (MMD).^1^, ^2^, ^3^ This disease is also known as spontaneous steno‐occlusion of the circle of Willis and is characterized by arterial negative remodeling^4^ involving shrinkage of the arterial wall, resulting in a stenotic lumen instead of intraluminal plaque deposition.^5^ *RNF213* encodes a protein containing dual AAA+ ATPase domains and an E3 ligase domain, which plays an important role in regulating vascular remodeling and angiogenesis.^2^, ^6^

The single nucleotide polymorphism of *RNF213* was previously found in 0.9% to 2.7% of healthy individuals in an East Asian population.^2^, ^7^, ^8^ A recent large case‐control study of 46 958 Japanese participants demonstrated that the p.R4810K variant was a strong risk factor for ischemic stroke (IS) and was found in 5.2% of patients with IS and in 2.1% of healthy controls.^9^ Moreover, the variant was found in approximately 20% to 50% of the patients without MMD with anterior intracranial artery steno‐occlusion in East Asia.^3^, ^10^ Furthermore, a higher prevalence of extracranial systemic artery steno‐occlusion was observed in patients with several *RNF213* variants compared with noncarriers.^11^

Although the p.R4810K variant may mechanistically cause negative remodeling of intracranial and extracranial systemic arteries, its impact on cervical arteries is still unclear. Carotid ultrasonography (CUS) is the gold standard noninvasive modality for measuring cervical artery diameters.^12^, ^13^, ^14^ Therefore, the present study aimed to investigate the association of the p.R4810K variant with the outer diameter of cervical portions of carotid and vertebral arteries (VAs) in patients with IS using CUS.

## Methods

Anonymized data that support the findings of this study are available from the corresponding author upon reasonable request and after permission from the ethics committees.

### Study Design and Population

This prospective observational study was performed at the National Cerebral and Cardiovascular Center (NCVC), Suita, Japan. All patients with IS were retrospectively registered in the NCVC Stroke Registry,^15^, ^16^, ^17^ signed a comprehensive consent form for the NCVC biobank, and were genotyped for the presence of the *RNF213* p.4810K variant.

Patients enrolled in this registry from May 2015 to January 2019 were retrospectively reviewed, and those who met the following criteria were included in this study: (1) CUS data available for outer diameters of bilateral cervical arteries and (2) written informed consent provided for participation in the present study. Patients with definite or probable MMD based on diagnostic criteria were excluded.^18^ Patients with only the diameter of either cervical carotid artery were also excluded.

Clinical and radiological data were prospectively collected in a database and retrieved for retrospective analysis. The study was conducted in accordance with the Declaration of Helsinki standards and was approved by the Research Ethical Committee of NCVC and the NCVC Biobank. The NCVC Stroke Registry is registered with ClinicalTrials.gov (NCT02251665).

### *RNF213* Genotyping Examination

Genotyping of the *RNF213* p.R4810K variant was performed using a fully automated gene analysis system (GTS‐7000 [Shimadzu Corporation, Kyoto, Japan] or LightCycler 96 [Roche, Basel, Switzerland]). These systems allow the direct detection of single nucleotide polymorphisms from 1 μL of whole blood using real‐time polymerase chain reaction (PCR).^19^ The cycling program includes 5 minutes of preincubation at 95 °C followed by 35 2‐step amplification cycles at 95 °C for 5 seconds and 60 °C for 15 seconds. Primer sequences were the following: 5′‐TTCCAGAACGTCCAGCAAGT‐3′ (forward) and 5′‐ACAGTCCTGGTCCTGTCAGA‐3′ (reverse). The probe sets were the following: 5′‐CTCCATCAGAGGCTTCCT‐3′ and 5′‐CTCCATCAAAGGCTTCCT‐3′. The AA or GA variant for p.R4810K was used to define variant carriers, and the wild‐type homozygote GG genotype was used to define variant noncarriers.

### Clinical Data Collection

The following clinical data were collected: patient sex, age, atherosclerotic risk factors (hypertension [diagnosis at hospital discharge or use of antihypertensive medications before the index IS], diabetes [diagnosis at hospital discharge or on antidiabetic treatment before the index IS], and dyslipidemia [diagnosis at hospital discharge or on lipid‐lowering therapy before the index IS]), and current smoking status (just before hospital admission). Steno‐occlusion of the intracranial internal carotid artery (ICA), M1 segment of the middle cerebral artery (MCA), the intracranial VA, and basilar artery (BA) was evaluated using digital subtraction angiography, computed tomography angiography, and/or magnetic resonance (MR) angiography. The degree of intracranial artery stenosis (no or mild stenosis, 0%–49%; moderate, 50%–69%; or severe stenosis or occlusion, 70%–100%) was identified by vascular neurologists (E.Y. and T.Y.) who were blind to the clinical information; a joint assessment was carried out for consensus findings if required. We defined the percent stenosis of an intracranial artery based on a standardized method.^20^ Stroke subtype was determined according to the Trial of ORG 10172 in Acute Stroke Treatment criteria by vascular neurologists.^21^

### Echocardiographic Protocol and Target Vessel Assessment

**Figure 1:**
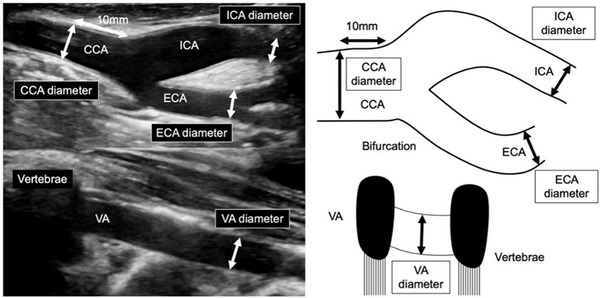
**Carotid ultrasonography measurement of cervical artery diameters**. CCA indicates common carotid artery; ECA, external carotid artery; ICA, internal carotid artery; and VA, vertebral artery.

**Figure 2:**
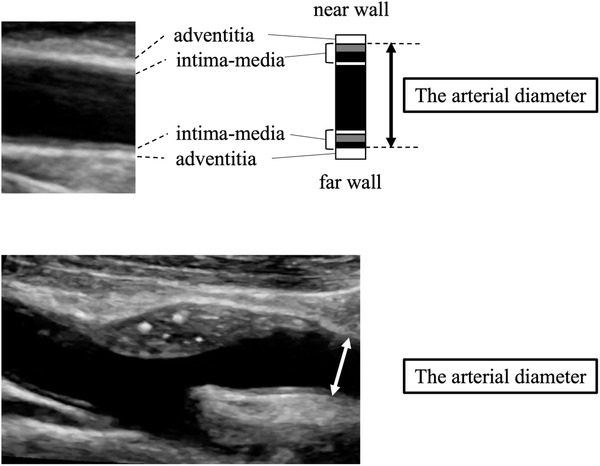
**Carotid ultrasonography measurement of arterial diameters**.

CUS was performed using LOGIQ E9 and LOGIQ S8 (GE Healthcare, Milwaukee, WI) and Aplio 500, Aplio MX, Aplio XG, and Aplio XV (Canon, Ohta‐ku, Tokyo, Japan) with a linear probe. The arterial diameter was defined as the distance between the inner adventitia in the near and far walls^13^, ^22^ using B‐mode imaging with a longitudinal view as shown in Figure 1 and according to the Japan Academy of Neurosonology guidelines.^13^ As shown in Figure 2, the arterial diameter at each point was measured as follows: common carotid arteries (CCAs) at 10 mm proximal from the carotid bulb, cervical VAs between any 2 of the third to the sixth cervical vertebrae, and cervical ICAs and external carotid arteries at a distal point where the diameter was stable. The current study used averaged diameters of both sides of CCAs, cervical ICAs, and external carotid arteries as described previously^22^ and the diameter of dominant cervical VAs, defined as the larger diameter of bilateral VAs. Because few previous studies have assessed the diameter of cervical VAs, we devised a method of evaluation using the dominant diameter of VAs considering anatomical asymmetrical variations caused by unilateral hypoplasia or termination into the posterior inferior cerebellar artery. Such asymmetry and termination into the posterior inferior cerebellar artery result in a smaller cervical VA diameter^23^, ^24^ and a lack of communication with the BA or intracranial artery.^24^

### Statistical Analysis

Data are summarized as mean±SD or median (interquartile range) values for continuous variables and as frequencies and percentages for categorical variables. Statistical differences between variant and nonvariant carriers were assessed using the Mann–Whitney *U* test or the χ^2^ test as appropriate. The baseline background and the diameter of the target cervical arteries were compared between variant carriers and noncarriers by univariate analysis. Logistic regression models were constructed, and odds ratios (ORs) with 95% CIs for *RNF213* p.R4810K variant carriers were calculated using noncarriers as a reference. Model 1 included variant carriers plus patient age and sex. Model 2 included variant carriers plus variables showing significant differences in univariate analysis.

The sensitivity analysis was restricted to patients without bilateral steno‐occlusion in target vessels or arteries distal to the target vessels because the presence or absence of intracranial artery steno‐occlusion can impact the reduction of the diameter of cervical arteries.^25^, ^26^ Steno‐occlusion of the cervical ICA was defined as a peak systolic velocity >200 cm/second or occlusion,^27^ and steno‐occlusion of the M1 segment of the MCA was defined as >70% stenosis according to a standardized method or occlusion by MR angiography.^20^ The patient background and mean diameters of the CCA and cervical ICA, external carotid artery, and VA were compared between variant carriers and noncarriers because steno‐occlusion of any vessels distal to the target vessels affects evaluation of the target vessel diameter.^28^, ^29^, ^30^ When the target vessels were the CCA or cervical ICA, the distal vessels corresponded to intracranial ICA or the M1 segment of the MCA, and when the target vessel was the cervical VA, the distal vessels corresponded to intracranial VA or BA. When unilateral cervical or distal intracranial moderate/severe steno‐occlusion was observed, the diameter of the nonaffected artery alone was used for analysis without averaging the bilateral arteries. In the case of steno‐occlusion of the BA, cervical VA was not subject to evaluation.

All reported *P* values were 2‐tailed, with the level of statistical significance set at *P*<0.05. All statistical analyses were performed using R software version 3.6.1 (R Foundation for Statistical Computing, Vienna, Austria).

## Results

### Patient Characteristics

**Figure 3:**
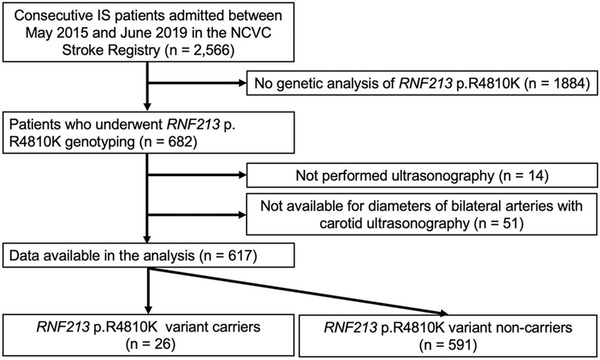
**Study flowchart**. IS indicates ischemic stroke; NCVC, National Cerebral and Cardiovascular Center; and *RNF213*, ring finger protein 213.

The study flowchart is shown in Figure 3. Of the 2566 patients with acute IS during the study period, 682 (18.0%) underwent genetic analysis of *RNF213* p.R4810K. Of these, 65 were excluded because of the absence of bilateral target artery diameters obtained by CUS. Among the analyzed data for 617 patients (204 women [33.1%]; median age, 74 years [interquartile range, 66–81]), the *RNF213* p.R4810K variant was found in 26 (4.2%). Steno‐occlusion of the intracranial arteries was assessed by digital subtraction angiography in 11.8% of patients, computed tomography angiography in 3.4%, and MR angiography in 84.8%. Patients with moyamoya vasculopathy (quasi‐MMD or akin‐MMD) were not included.

Variant carriers were significantly younger (67 versus 74 years; *P*<0.01) and significantly more likely to have steno‐occlusion of the M1 segment of the MCA (38.5% versus 6.9%; *P*<0.01) and large artery atherosclerosis (53.8% versus 22.0%; *P*<0.01) than noncarriers. No significant difference was seen in the medical histories between the 2 groups. Variant carriers had significantly smaller mean outer diameters in the CCA (7.25 versus 8.22 mm; *P*<0.01), cervical ICA (4.99 versus 5.55 mm; *P*<0.01), and cervical VA (3.55 versus 4.10 mm; *P*<0.01), but not in the cervical external carotid artery (3.86 versus 4.22 mm; *P*=0.34), than noncarriers. Patient data are shown in Table 1. Multivariable logistic regression analysis revealed a significant association between the *RNF213* p.R4810K variant and the mean diameter of CCA (per 1‐mm decrease: adjusted OR, 2.94; 95% CI, 1.69–5.00; *P*<0.01), cervical ICA (adjusted OR, 1.67; 95% CI, 1.03–2.63; *P*=0.03), and cervical VA (adjusted OR, 2.63; 95% CI, 1.40–5.00; *P*<0.01; Table 2).

**Table 1:** Patient Data

		*RNF213* p.R4810K variant^*^		
	All, N=617	Carriers, n=26	Noncarriers, n=591	*P* value
Female sex	204 (33.1)	11 (42.3)	193 (32.7)	0.42
Age, y	74 (66–81)	67 (56–74)	74 (67–81)	<0.01
Medical history				
Hypertension	485 (78.6)	19 (73.1)	466 (78.9)	0.65
Dyslipidemia	398 (64.5)	13 (50.0)	385 (65.1)	0.17
Diabetes	159 (25.8)	4 (15.4)	156 (26.4)	0.31
Current smoking	196 (31.2)	9 (34.6)	187 (31.6)	0.92
Arterial steno‐occlusion^*^				
Intracranial ICA	32 (5.2)	4 (15.4)	28 (4.8)	0.05
Cervical ICA	21 (3.4)	3 (11.5)	18 (3.0)	0.07
M1 segment of the MCA	51 (8.3)	10 (38.5)	41 (6.9)	<0.01
BA	10 (1.6)	0 (0.0)	10 (1.7)	1.00
Intracranial VA	22 (3.6)	0 (0.0)	22 (3.7)	0.64
Target carotid artery diameters				
CCA, mm	8.18±0.98	7.25±1.16	8.22±0.95	<0.01
Cervical ICA, mm	5.53±0.98	4.99±1.09	5.55±0.97	<0.01
ECA, mm	4.21±1.23	3.86±0.90	4.22±1.24	0.34
Cervical VA, mm	4.07±0.70	3.55±0.66	4.10±0.69	<0.01
Stroke subtypes				
Large artery atherosclerosis	138 (22.4)	14 (53.8)	124 (21.0)	<0.01
Cardioembolism	167 (27.1)	6 (23.1)	161 (27.2)	0.81
Small vessel occlusion	150 (24.3)	3 (11.5)	147 (24.9)	0.19
Others/undetermined	156 (25.3)	2 (7.7)	154 (26.0)	0.06

Data are presented as number (percentage), median (interquartile range), or mean±SD. BA indicates basilar artery; CCA, common carotid artery; ECA, external carotid artery; ICA, internal carotid artery; MCA, middle cerebral artery; MMD, moyamoya disease; *RNF213*, ring finger protein 213; and VA, vertebral artery.

^*^Patients with definite or probable MMD based on diagnostic criteria, including moyamoya vasculopathy (quasi‐MMD or akin‐MMD), were excluded.

**Table 2:** Logistic Regression Analyses for Cervical Arteries

Target carotid artery	Crude OR^*^ (95% CI)	*P* value	Adjusted OR (95% CI), model 1^†^	*P* value	Adjusted OR (95% CI), model 2^‡^	*P* value
CCA, per 1‐mm decrease	3.23 (2.00–5.26)	<0.01	2.94 (1.69–5.00)	<0.01	2.63 (1.56–4.35)	<0.01
Cervical ICA, per 1‐mm decrease	2.04 (1.25–3.33)	<0.01	1.66 (1.03–2.70)	0.03	1.67 (1.03–2.63)	0.03
Cervical ECA, per 1‐mm decrease	1.23 (0.79–1.92)	0.36	1.15 (0.70–1.89)	0.58	1.16 (0.72–1.85)	0.55
Cervical VA, per 1‐mm decrease	3.13 (1.69–5.56)	<0.01	2.56 (1.33–4.76)	<0.01	2.63 (1.40–5.00)	<0.01

CCA indicates common carotid artery; ECA, external carotid artery; ICA, internal carotid artery; OR, odds ratio; *RNF213*, ring finger protein 213; and VA, vertebral artery.

^*^ORs with 95% CIs for *RNF213* p.R4810K variant carriers were calculated using noncarriers as a reference.

^†^ Models for carotid arteries adjusted for age and sex.

^‡^ Models for carotid arteries adjusted for age and large artery atherosclerosis.

Baseline characteristics and CUS data of cervical arteries proximal to the anterior or posterior circulation were compared between variant carriers and noncarriers for subgroup analysis in Tables S1 and S2 in the Data Supplement. In the sensitivity analysis of vessels responsible for anterior circulation, 114 patients with bilateral steno‐occlusion in the cervical/intracranial ICA or M1 segment of the MCA were excluded. Variant carriers (n=22) had a significantly smaller mean diameter of the CCA (7.25 versus 8.28 mm; adjusted OR, 3.44; 95% CI, 2.08–5.88; *P*<0.01) and cervical ICA (4.94 versus 5.54 mm; adjusted OR, 2.04; 95% CI, 1.23–3.33; *P*<0.01) than noncarriers (n=481). In the sensitivity analysis of cervical VA, 29 patients with steno‐occlusion in the bilateral cervical/intracranial VA or BA were excluded. Variant carriers (n=26) also had a significantly smaller mean diameter of cervical VA (3.55 versus 4.11 mm; adjusted OR, 3.23; 95% CI, 1.72–5.88; *P*<0.01) than noncarriers (n=562; Table S3 in the Data Supplement).

## Discussion

The major finding of the present study is that among patients with IS, the outer diameters of the CCA, cervical ICA, and VA were significantly narrowed in patients carrying the *RNF213* p.R4810K (c.14429G>A) variant compared with noncarriers. The variant was also found to affect the outer diameters of these cervical arteries when patients with bilateral steno‐occlusion in target vessels or arteries distal to the target vessels were excluded. Therefore, this variant may affect not only intracranial arteries but also extracranial cervical arteries regardless of anterior or posterior circulation, leading to systemic structural changes characterized by a decreased outer diameter. Nevertheless, differences in background features (particularly age and the frequency of large artery atherosclerosis) between carriers and noncarriers should be considered when interpreting the present results. To resolve these biases, we designed an adjusted model with the factors associated with outcomes.

Patients with MMD have progressive steno‐occlusion caused by negative remodeling in the terminal portion of the intracranial ICA.^4^ Moreover, patients with IS with the *RNF213* p.R4810K variant have smaller outer vessel diameters in the intracranial ICA or proximal MCA^31^ and in the contralateral (nonaffected) MCA^10^ compared with those without the variant. Embryologically, both intracranial and extracranial ICA originate from primitive ICAs, so developmental vascular abnormalities underlying MMD can cause not only intracranial ICA stenosis but also cervical ICA stenosis.^32^ In fact, the cervical ICA diameter on the nonaffected side was previously shown to be smaller in patients with unilateral MMD than in the healthy volunteer population.^33^ These earlier reports are consistent with our findings that the diameters of cervical arteries were significantly narrowed in patients with IS carrying the p.R4810K variant compared with noncarriers. Interestingly, our study showed no significant difference in sex between the *RNF213* p.R4810K variant carriers and noncarriers among patients with IS, although research has shown that female carriers are generally 2 times more likely to develop MMD than male carriers.^34^ The p.R4810K variant carriers in the current study comprised 15 male and 11 female patients, which differs from the general male:female ratio of 1:2–3 among patients with MMD. However, because patients with MMD were excluded and the number of variant carriers was limited in this study, further research is required to determine how sex affects the cervical artery diameters.

Another intriguing finding in our study is that the *RNF213* p.R4810K variant was associated with narrowing of the cervical VA, which is responsible for posterior circulation. Kamimura et al^35^ reported that the p.R4810K variant was rarely associated with intracranial artery stenosis in the posterior circulation. However, a recent East Asian study used MR angiography to show that p.R4810K affected the diameters of intracranial arteries in both the anterior and posterior circulations.^30^, ^36^ Furthermore, an observational study from South Korea revealed an association between the *RNF213* p.R4810K variant and extracranial systemic artery stenosis.^11^ Based on the aforementioned findings, the *RNF213* p.R4810K variant may affect systemic arteries including anterior and posterior intracranial arteries, extracranial cervical arteries including cervical VAs, and extracranial systemic arteries. Our results showed that p.R4810K affected the cervical VA diameter, but further studies are needed to investigate whether there is an association between changes in intracranial arterial diameters in the posterior circulation and p.R4810K.

Plausible reasons for an association between p.R4810K and the narrowing of cervical arteries include the fact that the variant can affect systemic arteries. First, several studies showed that patients heterozygous for p.R4810K had classical MMD, whereas homozygotes had systemic vascular diseases in the aorta and renal, celiac, iliofemoral, and peripheral pulmonary arteries.^11^, ^37^, ^38^ This is in line with the new disease concept of *RNF213*‐related vasculopathy^29^, ^38^, ^39^, ^40^, ^41^ and, generally, neurocristopathy^6^, ^42^, ^43^ (developmental abnormalities of neural crest‐derived cells). Second, in vitro and in vivo experiments showed that the ubiquitously expressed RNF213 protein is associated with angiogenesis and vascular inflammation.^44^ Third, caveolin‐1, which is implicated in MMD, is a protein scaffold of the caveolae plasma membrane associated with mechanotransduction in vascular physiology and atherogenesis.^45^, ^46^

There are some limitations to the present study. First, it only enrolled Japanese participants, so extrapolations to other ethnic groups should be made with caution, demonstrating the need for multinational validation studies. We detected the variant in 4.2% of patients, which is higher than the frequency reported previously.^2^, ^7^, ^8^ Second, this was a retrospective study with a potential risk of selection bias. Indeed, only 27% (682/2566) of all patients with IS underwent *RNF213* p.R4810K genotyping because of difficulties in providing informed consent caused by issues with old age, impaired consciousness, cognitive impairment, and advanced frailty. The relatively low rate of RNF213 p.R4810K genotyping may have resulted in selection bias. Third, several variables were unmeasured, including patient height, weight, obesity, body surface area, and neck length. These should be considered in future studies for their possible correlation with CCA and ICA diameters obtained by CUS. Fourth, the small sample size did not allow us to undertake multivariate analyses by adjusting for all clinically relevant factors simultaneously. Fifth, the fact that MR angiography, which is much less reliable for stenosis grading, was used to evaluate the intracranial arteries in the sensitivity analysis may have resulted in overestimation or underestimation of the stenosis rate of the intracranial arteries.^47^, ^48^ Finally, all patients had a history of IS, so the effect of the *RNF213* p.R4810K variant on the outer diameter of cervical arteries should be examined in a population‐based cohort in any future study.

## Conclusions

Adult patients with IS with the *RNF213* p.R4810K variant were observed to have significantly smaller outer diameters of cervical arteries in both anterior and posterior circulations. Further studies are warranted to comprehend and define the novel disease spectrum of *RNF213*‐related vasculopathy in the East Asian population.

## Sources of Funding

This study was supported by the Japan Agency for Medical Research and Development and the SENSHIN Medical Research Foundation.

## Disclosures

Dr Yoshimoto reports lecture's fees from Takeda Pharmaceutical and Nippon Boehringer Ingelheim outside the submitted work. Dr Washida reports lecturer's fees from Bayer Yakuhin and Daiichi Sankyo outside the submitted work. Dr Koga reports honoraria from Bayer Yakuhin and Daiichi‐Sankyo; scientific advisory board compensation from Ono; and research support from Takeda, Daiichi‐Sankyo, Nippon Boehringer Ingelheim, Astellas, and Shionogi outside the submitted work. Dr Toyoda reports lecturer's fees from Daiichi Sankyo, Otsuka, Novertis, Abbott Medical, Bayer, and Bristol‐Myers Squibb outside the submitted work. Dr Ihara reports lecturer's fees from Daiichi Sankyo and Eisai and grant support from Panasonic, GE Precision Healthcare LLC, Bristol‐Myers Squibb, and Shimadzu Corporation outside the submitted work. The other authors report no conflicts.

## Supporting information

Supporting Information
