# Supplementary material for: Association of the RNF213 p.R4810K Variant With the Outer Diameter of Cervical Arteries in Patients With Ischemic Stroke
Source: Stroke Vasc Interv Neurol. 2022 Mar 24;2(6):e000298. doi: 10.1161/SVIN.121.000298 (PMC12778827; doi:10.1161/SVIN.121.000298)
Supplement: Supplementary file 1 — Supporting Information [file SVI2-2-e000298-s001.pdf]

SUPPLEMENTAL MATERIAL

Table I. Baseline data and anterior circulation CUS findings between variant carriers and non-carriers for subgroup analysis. (Page 2)

Table II. Baseline data and posterior circulation CUS findings between variant carriers and non-carriers for subgroup analysis. (Page 3)

Table III. Logistic regression analyses for subgroup analysis. (Page 4)

Table I. Baseline data and anterior circulation CUS findings between variant carriers and non-carriers for subgroup analysis.

	Without bilateral moderate/severe stenosis or occlusion in the cervical/intracranial ICA or M1 segment of the MCA		
	RNF213 **p.R4810K (c.14429G>A) variant**		***P*-value**
	**Carriers (n = 22)**	**Non-carriers (n = 481)**	
Female	10 (45.5)	161 (33.5)	0.35
Age, years (range)	68 [57.5–74.0]	74 [66.0–81.0]	0.01
**Medical history**			
Hypertension	16 (72.7)	383 (79.6)	0.61
Dyslipidemia	11 (50.0)	312 (64.9)	0.23
Diabetes mellites	2 (9.1)	123 (25.6)	0.13
Current smoking	8 (36.4)	172 (35.8)	1.00
**Target carotid artery diameters**			
CCA, mm	7.25 ± 1.18	8.28 ± 0.94	<0.01
Cervical ICA, mm	4.94 ± 1.30	5.54 ± 1.00	<0.01
Cervical ECA, mm	3.88 ± 0.92	4.27 ± 1.17	0.27
**Stroke subtype**			
Large-artery atherosclerosis	11 (50.0)	87 (18.1)	<0.01
Cardioembolism	5 (22.7)	156 (32.4)	0.47
Small-vessel occlusion	3 (13.6)	123 (25.6)	0.31
Others/undetermined	2 (9.1)	111 (23.1)	0.20

Data are presented as number (%), median [interquartile range] or mean (standard deviation).

CCA, common carotid artery; CUS, carotid ultrasonography; ECA, external carotid artery; ICA, internal carotid artery; MCA, middle cerebral artery.

Table II. Baseline data and posterior circulation CUS findings between variant carriers and non-carriers for subgroup analysis.

	**Without moderate/severe stenosis or occlusion in the bilateral cervical/intracranial VA or BA**		
	***RNF213* p.R4810K (c.14429G>A) variant**		***P*-value**
	**Carriers (n = 26)**	**Non-carriers (n = 562)**	
Female	11 (42.3)	183 (32.6)	0.41
Age, years (range)	67 [55.5–74.0]	74 [67.0–81.0]	<0.01
**Medical history**			
Hypertension	19 (73.1)	446 (79.4)	0.60
Dyslipidemia	13 (50.0)	368 (65.5)	0.16
Diabetes mellites	4 (15.4)	144 (25.6)	0.35
Current smoking	9 (34.6)	179 (31.9)	0.94
**Target carotid artery diameters**			
Cervical VA, mm	3.55 ± 0.66	4.11 ± 0.69	<0.01
**Stroke subtype**			
Large-artery atherosclerosis	14 (53.8)	117 (20.8)	<0.01
Cardioembolism	6 (23.1)	129 (38.5)	0.80
Small-vessel occlusion	3 (11.5)	141 (25.1)	0.18
Others/undetermined	2 (7.7)	145 (25.8)	0.06

Data are presented as number (%), median [interquartile range] or mean (standard deviation).

BA, basilar artery; CUS, carotid ultrasonography; VA, vertebral artery.

Table III. Logistic regression analyses for subgroup analysis.

Target carotid artery	Crude OR (95% CI)	*P*-value	Adjusted OR (95% CI)*, Model 1†	*P*-value	Adjusted OR (95% CI), Model 2‡	*P*-value
CCA (/decreased by 1 mm)	3.44 (2.08–5.88)	<0.01	3.33 (1.85–6.25)	<0.01	3.03 (1.72–5.26)	<0.01
Cervical ICA (/decreased by 1 mm)	2.04 (1.23–3.33)	<0.01	1.72 (1.03–2.86)	0.03	1.67 (1.01–2.70)	0.03
Cervical ECA (/decreased by 1 mm)	1.31 (1.23–3.33)	0.28	1.21 (0.71–2.08)	0.47	1.28 (0.77–2.13)	0.34
Cervical VA (/decreased by 1 mm)	3.23 (1.72–5.88)	<0.01	2.63 (1.35–5.00)	<0.01	2.63 (1.39–5.00)	<0.01

* ORs with 95% CIs for *RNF213* p.R4810K variant carriers were calculated using non-carriers as a reference.

† Model 1 for carotid arteries adjusted for age and sex.

‡ Model 2 for carotid arteries adjusted for age and large-artery atherosclerosis.

CCA, common carotid artery; CI, confidence interval; CUS, carotid ultrasonography; ECA, external carotid artery; ICA, internal carotid artery; MCA, middle cerebral artery; OR, odds ratio; VA, vertebral artery
